# Development and Validation of a Nomogram for Predicting the Risk of Adverse Cardiovascular Events in Patients with Coronary Artery Ectasia

**DOI:** 10.3390/jcdd8120186

**Published:** 2021-12-14

**Authors:** Zhongxing Cai, Yintang Wang, Luqi Li, Haoyu Wang, Chenxi Song, Dong Yin, Weihua Song, Kefei Dou

**Affiliations:** 1 Cardiometabolic Medicine Center, Department of Cardiology, Fuwai Hospital, State Key Laboratory of Cardiovascular Disease, National Center for Cardiovascular Diseases, Chinese Academy of Medical Sciences and Peking Union Medical College, Beijing 100037, China; caizhongxing@fuwai.com (Z.C.); wanghaoyu@fuwai.com (H.W.); songchenxi@fuwai.com (C.S.); yindong@fuwai.com (D.Y.); 2 Department of Cardiology, Beijing Tsinghua Changgung Hospital, School of Clinical Medicine, Tsinghua University, Beijing 102218, China; wyta02494@btch.edu.cn; 3 Institute of Medical Information, Chinese Academy of Medical Sciences and Peking Union Medical College, Beijing 100020, China; li.luqi@imicams.ac.cn

**Keywords:** coronary artery ectasia, cardiovascular death, myocardial infarction, prediction model

## Abstract

Coronary artery ectasia (CAE) is a rare finding and is associated with poor clinical outcomes. However, prognostic factors are not well studied and no prognostication tool is available. In a derivation set comprising 729 consecutive CAE patients between January 2009 and June 2014, a nomogram was developed using Cox regression. Total of 399 patients from July 2014 to December 2015 formed the validation set. The primary outcome was 5-year major adverse cardiovascular events (MACE), a component of cardiovascular death and nonfatal myocardial infarction. Besides the clinical factors, we used quantitative coronary angiography (QCA) and defined QCA classification of four types, according to max diameter (< or ≥5 mm) and max length ratio (ratio of lesion length to vessel length, < or ≥1/3) of the dilated lesion. A total of 27 cardiovascular deaths and 41 nonfatal myocardial infarctions occurred at 5-year follow-up. The nomogram effectively predicted 5-year MACE risk using predictors including age, prior PCI, high sensitivity C-reactive protein, N-terminal pro-brain natriuretic peptide, and QCA classification (area under curve [AUC] 0.75, 95% CI 0.68–0.82 in the derivation set; AUC 0.71, 95% CI 0.56–0.86 in the validation set). Patients were classified as high-risk if prognostic scores were ≥155 and the Kaplan–Meier curves were well separated (log-rank *p* < 0.001 in both sets). Calibration curve and Hosmer–Lemeshow test indicated similarity between predicted and actual 5-year MACE survival (*p* = 0.90 in the derivation and *p* = 0.47 in the validation set). This study developed and validated a simple-to-use method for assessing 5-year MACE risk in patients with CAE.

## 1. Introduction

Coronary artery ectasia (CAE) is defined as coronary artery dilation of at least 1.5 times the adjacent normal segment [1]. It is a rare finding in coronary angiography with reported incidence ranging from 0.3% to 5% [2]. CAE was reported to be associated with poor clinical outcomes. A retrospective study in 2017 indicated CAE was associated with an increased risk of cardiac death, nonfatal myocardial infarction (MI) in patients with acute myocardial infarction [3]. Another study demonstrated that CAE was an independent predictor of mortality [4]. However, prognostic factors of adverse cardiovascular events in patients with CAE were not well studied.

In the past years, risk prediction models were widely used in coronary diseases for risk stratification. For example, the SYNTAX score focused on the anatomy of coronary vasculature and is currently recommended to guide revascularization therapy in coronary artery disease (CAD) [5,6]. A combination of angiographical and clinical factors achieved better prediction [7]. However, no risk prediction model for CAE has been published by far. Previously our cohort study demonstrated that diffuse dilation is associated with poor long-term outcomes in patients with CAE [8], indicating that angiographical characteristics of dilated vessels might be important for risk prediction in these patients.

The study aimed to develop and validate a prognostic nomogram for patients with coronary artery ectasia, based on baseline angiographical characteristics and clinical factors, to help high-risk patient identification and clinical decision-making.

## 2. Materials and Methods

### 2.1. Study Population

**Figure 1:**
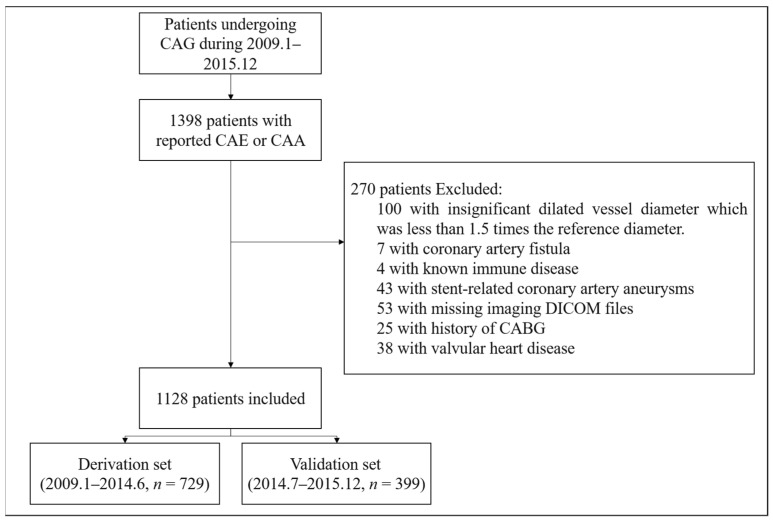
Flowchart of the study objects. CAE: coronary artery ectasia. CAA: coronary artery aneurysm. CAG: coronary angiography.

A total of 1128 patients were included and the flowchart of the study objects is shown in Figure 1. The derivation set comprised 729 consecutive CAE patients identified by coronary angiography from January 2009 to June 2014 in Fuwai hospital, Beijing. The validation set comprised 399 consecutive patients from July 2014 to December 2015. The angiographic criteria of CAE were defined as: (1) Abnormal dilation of more than 1.5-fold the diameter of adjacent normal segments; or (2) if there was no adjacent normal segment found, normal values of the corresponding segment from data in age-sex matched patients with normal coronary angiography were used as reference diameters [3,9] (Table S1 in the Supplementary Materials). The angiogram of each patient was screened by two experienced interventional cardiologists. The exclusion criteria were (1) Insignificant dilated vessel diameter which was less than 1.5 times the reference diameter; (2) coronary artery fistula; (3) stent-related coronary artery aneurysms; (4) known autoimmune disease; (5) missing DICOM format imaging files; (6) valvular heart disease; or (7) history of CABG. This study was approved by the Ethics Committee of Fuwai hospital. The study was performed in accordance with the Declaration of Helsinki. All eligible patients provided informed consent for long-term follow-up by telephone or clinic visit.

### 2.2. Clinical Data Collection

Medical records including medical history, laboratory tests, and echocardiography results were obtained from the hospital’s electronic medical records system. CAD risk factors queried included age, sex, current smoker, hypertension, diabetes, dyslipidemia, peripheral arterial disease, and family history of CAD. Details of the collected information are shown in Table S2 in the Supplementary Materials. The modified Modification of Diet in Renal Disease (MDRD) equations based on Chinese patients were applied to calculate the estimated glomerular filtration rate(eGFR) [10]. All laboratory tests were at baseline and before coronary angiography.

### 2.3. Angiographic Evaluation and Quantitative Coronary Angiography (QCA)

**Figure 2:**
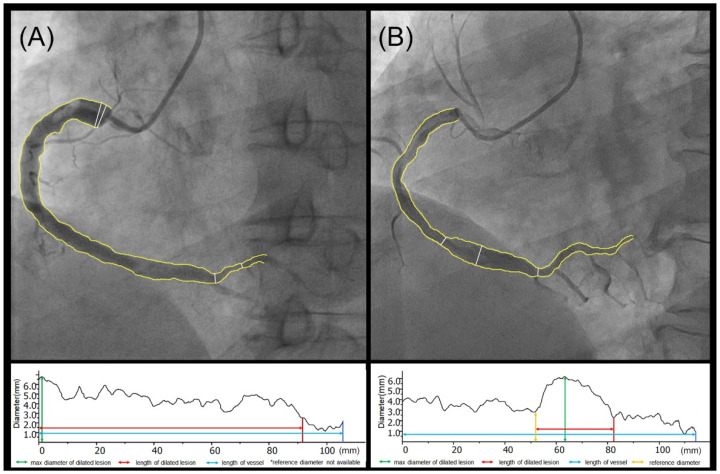
Instruction for QCA measurement. Examples for QCA measurement of a diffuse dilated lesion with no adjacent normal segment (**A**) and a focal dilated lesion with adjacent normal segment (**B**).

The DICOM format files of baseline coronary angiography were analyzed with Qangio XA version 7.3 (Medis, Leiden, The Netherlands) by an independent catheterization core laboratory. The definition of the coronary tree segments is as same as the SYNTAX score system [5]. Each dilated lesion was measured for its involved segment, max diameter, reference diameter, lesion length, and vessel length (Figure 2A,B). Angiographic features of the dilated coronary segments including contrast agent stasis, calcification, and thrombus were documented. Based on the above measurement, the maximum diameter of the dilated lesion (MAXD), maximum diameter dilation ratio (i.e., the ratio of max diameter to reference diameter, MAXD ratio), maximum length of the dilated lesion (MAXL), and maximum length ratio (i.e., the ratio of lesion length to vessel length, MAXL ratio) were calculated at patient level. Patients with MAXL ratio ≥ 1/3 were classified as diffuse dilation group and MAXL ratio < 1/3 was focal [11,12]. CAE with MAXD ≥ 5 mm was classified as large and MAXD < 5 mm was small [13]. To handle the correlation between MAXL ratio and MAXD (Table S3 in the Supplementary Materials), a new categorical variable “QCA characteristic classification” was generated: Type 1, MAXD < 5 mm & MAXL ratio < 1/3; Type2, MAXD < 5 mm & MAXL ratio ≥ 1/3; Type3, MAXD ≥ 5 mm & MAXL ratio < 1/3; Type4, MAXD ≥ 5 mm & MAXL ratio ≥ 1/3. The widely used Markis classification of CAE was also assessed [14]. Baseline SYNTAX scores of each patient were calculated to quantify the severity of combined coronary heart disease [5].

### 2.4. Outcomes and Follow-Up

Follow-up was conducted annually by telephone interviewers using standardized questionnaires. The primary outcome was 5-year major adverse cardiovascular events (MACE), which was a component of cardiovascular death and nonfatal MI. 1-year and 3-year MACE were also assessed. Notably, perioperative myocardial infarction was not included.

### 2.5. Statistical Analysis

Normally distributed continuous variables were expressed as mean ± standard deviation and compared using the *t*-test. Continuous data with non-normal distribution were summarized as median (interquartile range, IQR) and compared using the Mann–Whitney test. Categorical variables were expressed as counts (composition ratio), and compared using the Chi-square test or Fisher exact test as appropriate.

The overall survival nomogram was developed from a multivariable Cox regression model in a derivation set. For variable selection, we performed univariable Cox regression at first and variables with *p*-value < 0.15 were reserved as candidate variables. Spearman’s rank correlation coefficient was used to evaluate the correlation of candidate variables. Then all-subset Cox regression based on Akaike Information Criterion (AIC) was applied and the model with minimum AIC value was selected as the prediction model. The proportional hazards assumption of this model was tested by Schoenfeld residual. A prognostic score was calculated by summing the number of risk points corresponding to each weighted covariate. Individuals were subsequently classified for risk of MACE by prognostic scores using cut-off value optimized by X-tile version 3.6.1 (Rimm Lab, Yale School of Medicine, New Haven, CT, USA) [15]. The nomogram was assessed by discrimination and calibration in both derivation set and validation set. Receiver operating characteristic curve was performed and area under curve (AUC) was measured. Model performance was further examined through survival analysis using Kaplan Meier curves, and a wider separation in the curves indicated better discrimination. Calibration plots compared the actual Kaplan Meier survival estimates with predicted MACE survival probabilities. Further calibration of the nomogram was evaluated using the Hosmer-Lemeshow goodness-of-fit test according to 10 risk groups. All analyses were performed with R version 4.1.0 (R Foundation for Statistical Computing, Vienna, Austria).

## 3. Results

### 3.1. Clinical Features and Characteristics

The reported incidence of CAE ranged from 0.83% to 1.36% from 2009 to 2015 (Figure S1 in the Supplementary Materials). Baseline characteristics of the derivation set and validation set were listed in Table 1. The 5-year follow-up rate was 95.34% and 95.49% respectively. Total of 51 MACE occurred in the derivation set, including 20 cardiovascular death and 31 nonfatal MI. In the validation set, 7 cardiovascular death and 10 nonfatal MI were observed. The incidences of dyslipidemia and family history of CAD were higher in the 399 individuals of the validation set.

### 3.2. Coronary Angiography Evaluation

Most patients with CAE were combined with CAD (90.53% in derivation set and 87.97% in validation set). The right coronary artery was the most common dilated vessel, followed by the left anterior descending and left circumflex artery. In the derivation set, there was a higher prevalence of diffuse dilation and a slightly lower incidence of left main dilation. In QCA measurement, individuals in the validation set were with slightly greater MAXD. Detailed coronary angiography evaluation is shown in Table 2.

### 3.3. Nomogram Prediction of MACE

Continuous variables including age, left ventricular ejection fraction (LVEF), left ventricular internal dimension (LVID), N-terminal pro-brain natriuretic peptide (NT-proBNP), MAXL ratio, and categorical variable including prior PCI, high sensitivity C-reactive protein (hsCRP) > 3 mg/L, diffuse dilation (MAXL ratio > 1/3), MAXD > 5 mm, D-dimer > 0.5 mg/L, and QCA characteristic classification were selected as candidate variables according to univariable cox regression (Appendix A Table A1).

**Figure 3:**
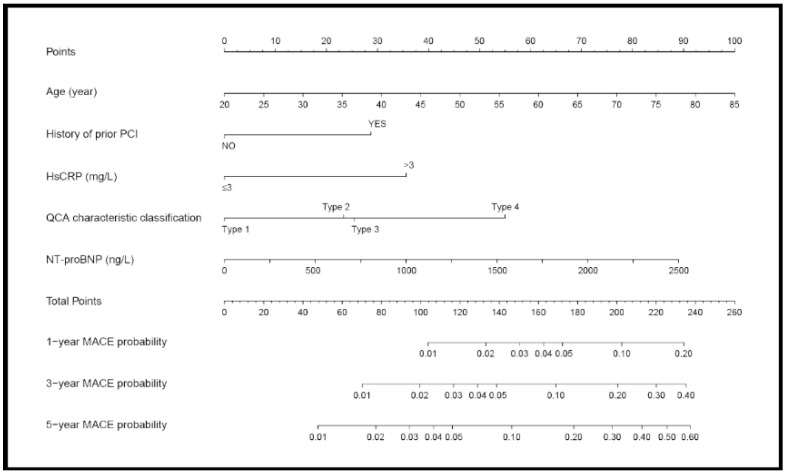
Nomogram for MACE prediction. Draw a perpendicular line from the corresponding axis of each risk factor until it reaches the top line labeled “Points”. Sum up the points of all risk factors then draw a line descending from the axis labeled “Total Points” until it intercepts each of the risk axes to determine 1-, 3-, and 5-year MACE probabilities.

**Figure A1:**
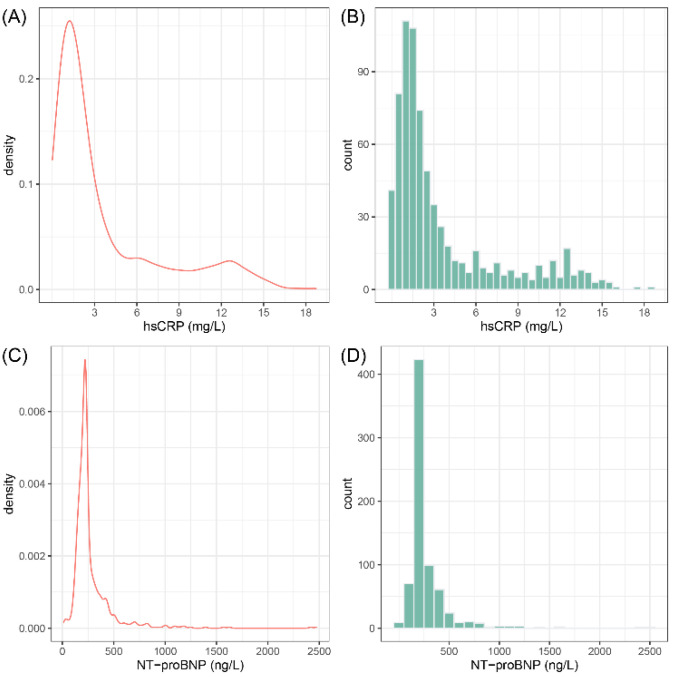
Density plots and histogram of hsCRP and NT-proBNP. hsCRP: (**A**,**B**). NT-proBNP: (**C**,**D**).

All subset Cox regression was carried out and the model with the lowest AIC was selected as the final prediction model, which contained five predictors including age, NT-proBNP, prior PCI, hsCRP > 3 mg/L, and QCA characteristic (Table 3). The distribution of hsCRP and NT-proBNP is shown in Figure A1 in the Appendix A. The proportional hazards assumption was tested by Schoenfeld residual (Figure S2 in the Supplementary Materials). Figure 3 displays the nomogram to predict the risk of MACE at 1-year, 3-year, and 5-year.

### 3.4. Validation of the Nomogram

**Figure 4:**
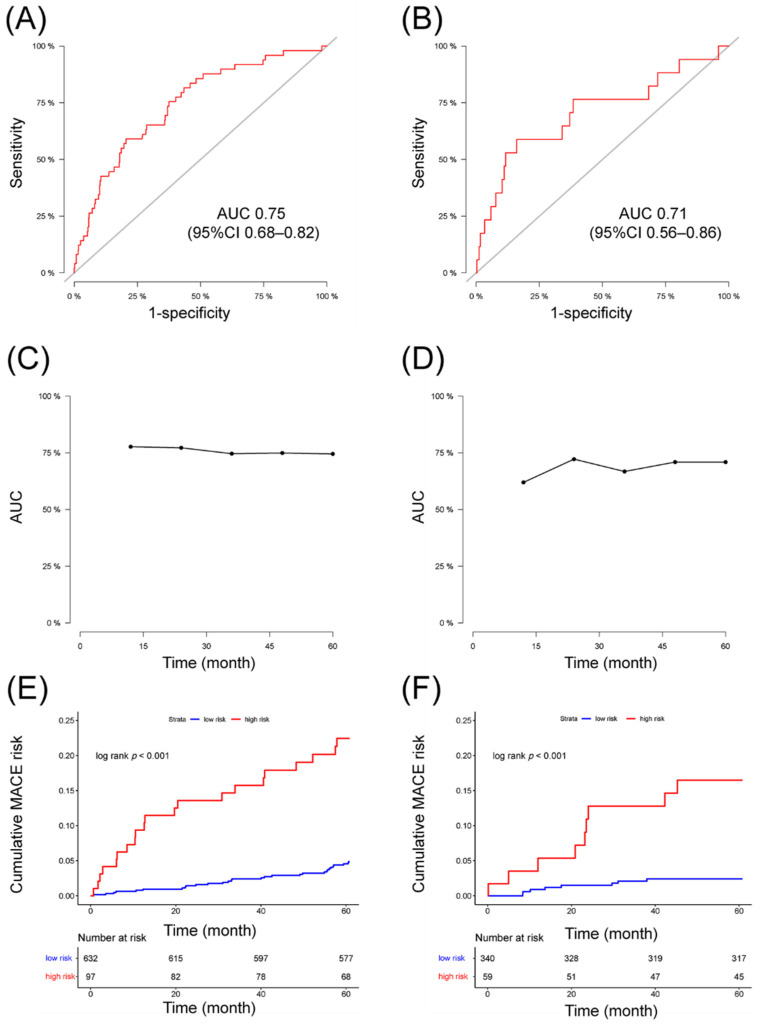
Discrimination of the nomogram in derivation and validation set. Receiver operating characteristic curve of 5-year MACE prediction in derivation set (**A**) and validation set (**B**). The area under curve of the nomogram for predicting 1-year to 5-year MACE in derivation data (**C**) and validation set (**D**). Kaplan–Meier curves of the derivation set (**E**) and validation set (**F**) stratified by prognostic scores. Low risk: prognostic score < 155. High risk: prognostic score ≥ 155.

**Figure 5:**
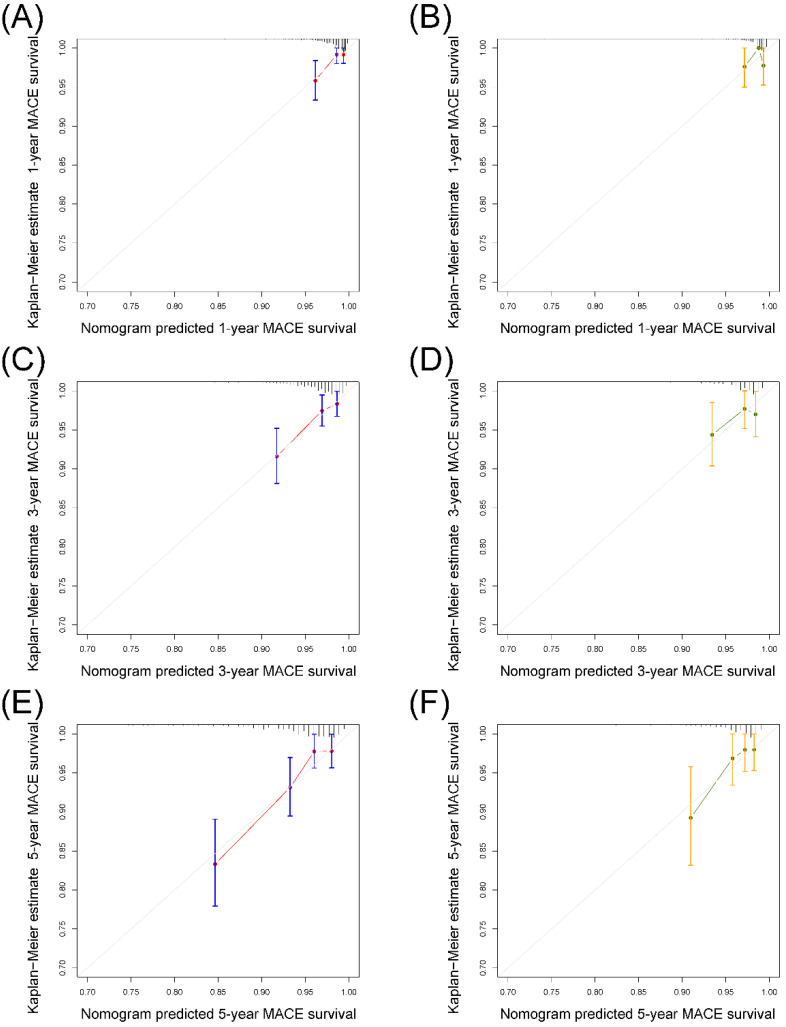
Calibration of the nomogram in derivation set and validation set. 1-year (**A**,**B**), 3-year (**C**,**D**) and 5-year (**E**,**F**) predictions. Data are from the derivation set (**A**,**C**,**E**) and validation set (**B**,**D**,**F**). This nomogram predicted cumulative incidence of MACE survival that were stratified in equally sized subgroups. For each subgroup, the average predicted MACE survival (nomogram-predicted MACE survival; *x*-axis) was plotted against the observed MACE survival Kaplan–Meier estimate MACE survival; *y*-axis). Vertical lines are 95% CIs of the MACE survival rate. Gray lines are the reference line, which would indicate where an ideal nomogram would lie.

AUC for prediction of 5-year MACE was 0.75 (95% CI 0.68–0.82) in derivation set and 0.71 (95% CI 0.56–0.86) in the validation set (Figure 4A,B). Additionally, the nomogram yielded AUC of 0.78, 0.77, 0.75, 0.75 for predicting 1-year to 4-year MACE risk in derivation set and 0.62, 0.72, 0.67, 0.71 in validation set (Figure 4C,D). Patients were divided into low-risk and high-risk groups using a cut-off prognostic score of 155. Kaplan–Meier curves for both datasets were reported in Figure 4E,F. The curves of the high-risk and the low-risk group appeared well separated, indicating reasonable discrimination. Figure 5 displayed calibration plots comparing predicted survival probabilities with actual Kaplan–Meier estimates in both sets. Hosmer–Lemeshow tests yielded chi-squares of 3.52 (*p* = 0.90) and 7.64 (*p* = 0.47) for the derivation and validation sets, respectively, indicating no significant difference between observed and predicted MACE survival in both datasets.

### 3.5. Severity of Coronary Stenosis and 5-Year MACE

Considering the potential prognostic role of coronary artery stenosis, we added the SYNTAX score directly to the final prediction model to assess if it was an independent predictor of the primary endpoint. However, in the multivariable Cox regression, the SYNTAX score was still not a predictive factor (HR = 1.008, 95% CI 0.98–1.037, *p* = 0.332) and had little effect on the model. Similarly, severe CAD, defined as the 3-vessel or LM disease, was also not a significant predictor (HR 1.228, 95% CI 0.69–2.19, *p* = 0.485).

## 4. Discussion

In this cohort of 1128 patients with CAE, we developed and validated a nomogram-illustrated prediction model for predicting 5-year MACE risk. This nomogram encompassed an extensive set of clinical risk factors that were easy to obtain, while also taking advantage of angiographically anatomical characteristics of coronary arteries measured by QCA. To our knowledge, this is the first risk prediction model for patients with CAE in a large cohort of this rare disease. It might be a valuable tool for clinical practice.

The definition of CAE in the current study was clearer than the classical definition, which simply defined CAE as coronary abnormal dilation of at least 1.5 times the adjacent normal segment. This classical definition might be confusing when there was no adjacent normal segment found due to extremely diffuse dilation or severe stenosis. Consistent with previous studies [3,9], we used corresponding segment diameter from data in age-sex matched patients with normal coronary angiography as a reference for such cases.

Nomograms have frequently been used in cancer prognosis in earlier times [16] and cardiovascular disease recently [17]. A nomogram is a visual and easy-to- use prognostic tool. In the current study, we considered cardiovascular death and nonfatal MI as primary outcomes. Previous studies indicated that coronary artery ectasia might be a systemic vascular disease, as it was associated with higher incidences of varicose veins [18] and increased risk of non-coronary adverse vascular events [19]. Consistently, there was a high incidence of CAE in patients with aortic aneurysms [20,21]. Therefore, death of cardiovascular disease, which included not only cardiac death but also other causes such as aortic aneurysm, was a reasonable outcome.

The validity of our nomogram was assessed by discrimination and calibration in two data sets. The AUC was more than 0.7 for predicting 5-year MACE risk in both derivation and validation set. The calibration curve showed great calibration for predicting 3-year and 5-year MACE in both sets. Hosmer–Lemeshow test was further performed and both sets were not significant, indicating suitable calibration of the prediction model. For 1-year and 3-year MACE prediction, AUC was 0.78 and 0.75 in derivation set but 0.62 and 0.67 in validation set, and the calibration curve showed the model might overestimate MACE risk in one subgroup in validation set. This might be a result of the limited sample size of the validation set and low cumulative event rate (<5% in all subgroups in the calibration curve) at 1 year.

Except for age, four additional variables including NT-proBNP, hsCRP ≥ 3 mg/L, history of prior PCI, and QCA characteristic classification were incorporated in the nomogram and provided new perspectives for us to understand this rare disease.

NT-proBNP is secreted from the cardiomyocytes into the circulation in response to cardiac stress [22]. It is closely related to cardiac function and regarded as a marker for the diagnosis and prognosis of heart failure [23]. In Kawasaki disease, which is a common cause of coronary aneurysms in teenagers, NT-proBNP is found to be a biomarker for diagnosis and cardiac systolic function [24,25]. However, less is known about its relationship with adverse cardiovascular events in patients with CAE. This study indicated that higher NT-proBNP levels were associated with a higher risk of MACE. Natriuretic peptides were also reported to be a marker to identify early cardiac target organ damage, such as asymptomatic myocardial ischemia, left ventricular diastolic, and systolic insufficiency [26], which might result in increased MACE risk.

HsCRP is an index of systemic inflammation. Our previous study demonstrated that a higher level of hsCRP > 3 mg/L was independently associated with cardiac death and nonfatal myocardial infarction in patients with CAE [27]. This time the association was confirmed in a larger population. About 3 mg/L was the upper limit of normal in our hospital’s laboratory, which was also the predictor of increased risk of coronary events and all-cause mortality in the general population [28]. Furthermore, the restricted cubic splines curve also indicated 3 mg/L was a reasonable cut-off value (Figure S3 in the Supplementary Materials). HsCRP was a known biomarker of worse clinical outcomes in patients with acute MI and a biomarker of residual inflammatory risk in cardiovascular disease [29,30,31]. This study suggested that inflammatory risk might be one of the pathophysiological mechanisms of CAE and played a role in the progression of this disease.

History of prior PCI was another predictor in the current study. Ectatic coronary artery with a large diameter was challenging for PCI [32]. One possible reason is that stent implantation in dilated coronary arteries might be more likely to result in stent malapposition. However, few optical coherence tomography or intravascular ultrasound study of CAE cohort has been reported, thus the incidence and clinical outcomes of acute and late stent malapposition in patients with CAE remained unknown. From another point, this factor contained information about coronary artery stenosis and atherosclerotic burden, as most prior PCIs were stenting indicated for coronary stenosis. Notably, we accessed SYNTAX score and severe CAD in both univariable Cox regression and multivariable regression but neither of them was a predictive factor in the current study. This is not to say that stenosis is not associated with prognosis, but to suggest that these variables such as the SYNTAX score may not be appropriate for assessing prognosis in patients with CAE.

The last predictor in the nomogram was QCA classification, a variable to access the severity and extent of the dilated lesion. Coronary artery ectasia was categorized as diffuse if the lesion involved more than a third of the artery length [11,12]. Our prior study indicated that diffuse coronary artery dilation was a predictor of poor long-term outcomes in patients with CAE [8]. The current QCA study further suggested that diameter ≥ 5 mm was associated with MACE in univariable Cox regression. Coronary aneurysms were classified as small empirically if diameter < 5 mm [13]. This study showed that 5 mm is not only a morphological cut-off value but also a potential predictor for clinical outcomes. However, due to the correlation between lesion length and diameter of the dilated vessel, that is, diffuse lesion tends to be larger in diameter, these two variables are not able to enter a multi-variable Cox regression model together. As noted earlier, we generated the predictor “QCA characteristic classification”, which combined the variable “diffuse” and “diameter ≥ 5 mm” to solve this problem. So both the factors were considered in the prediction model. This QCA study demonstrated again that coronary artery anatomy characteristic of dilated vessels was an important predictor of MACE in patients with CAE. In previous studies [33,34], empirical Markis classification of CAE was widely used but no association with mortality had been found. Consistently, it was not a significant risk factor in the current study and we proposed a new classification based on QCA which was shown to be a predictive factor of MACE.

Further still, we used Xtile [15] to generate optimal prognostic score cut-off value to stratify patients into low-risk and high-risk groups. A prognostic score < 155 was classified as low risk and ≥155 as high risk. The Kaplan–Meier curves were well separated according to the risk stratification. In addition to informing patients about their future MACE risk, the results may be used as guidance for intensive treatment strategies, such as anticoagulation therapy and strict risk factor control, for patients at high risks of MACE. Until now, the optimal medical therapy of CAE remained unknown, and high-risk subset patients might benefit from anticoagulation rather than antiplatelet [3]. However, comparative studies must be performed to assess the effect of intensive therapeutic strategies based on the current model.

### Study Limitations

First, although this model was assessed in derivation set and validation set, all participants were from a single-center and the limitation of a single-center cohort must be recognized. Second, the relatively small sample size might affect the effectiveness and evaluation of the model. Potential predictive variables might not be detected due to weakened statistical efficiency of the limited sample size. However, it must be emphasized that CAE was a rare finding and this current study comprising 1128 patients was the largest cohort in contemporary studies. Third, just like all other prediction models, the overall event rate might change as time goes by and treatment improves, thus a model might become inexact gradually. In the present study, patients in the validation set were from a later period and the MACE rate seemed to be relatively lower, but the nomogram still achieved good discrimination and calibration, indicating the reasonable clinical value of this model. Fourth, model performance is not extremely perfect, and there is room for improvement. Notably, intravascular imaging and coronary artery functional methods were not available in this study and they could provide more coronary artery physiological information, which might be important for risk prediction. Moreover, the primary end point of this study was at patient level, and studies aiming at vessel-oriented cardiac events (VOCEs) would provide more information. In the future, data from other centers are also required to access the current model in more external validation sets.

## 5. Conclusions

In the present analysis from a large consecutive cohort of patients with angiographically confirmed CAE, we proposed a nomogram for risk prediction. This nomogram, which consisted of QCA characteristic classification and four clinical variables including age, NT-proBNP, history of prior PCI, and increased hsCRP (>3 mg/L), offered clinicians a simple-to-use method for assessing 5-year MACE risk in patients with coronary artery ectasia.

## Figures and Tables

**Table 1:** Baseline characteristics of the derivation dataset and validation dataset.

	Derivation Set	Validation Set	*p*-Value
No. of patients	729	399	
Male	618 (84.77)	333 (83.46)	0.621
Age, yrs	57.30 ± 10.86	56.96 ± 11.25	0.627
BMI, kg/m^2^	26.42 [24.22, 28.70]	26.63 [24.44, 29.39]	0.272
Clinical presentation			0.175
Asymptomatic	13 (1.78)	11 (2.76)	
Stable angina	240 (32.92)	128 (32.08)	
Unstable angina	333 (45.68)	176 (44.11)	
NSTEMI	35 (4.80)	32 (8.02)	
STEMI	98 (13.44)	43 (10.78)	
Dyspnea	3 (0.41)	4 (1.00)	
Palpitation	7 (0.96)	5 (1.25)	
Prior MI	201 (27.52)	109 (27.32)	0.983
Prior PCI	177 (24.28)	97 (24.31)	1.000
Diabetes	168 (23.05)	95 (23.80)	0.829
Hypertension	483 (66.26)	267 (66.92)	0.873
Dyslipidemia	457 (62.69)	280 (70.18)	0.014
Peripheral arterial disease	12 (1.65)	10 (2.51)	0.439
Family history of CAD	103 (14.13)	92 (23.06)	<0.001
Current smoker	248 (34.02)	146 (36.59)	0.423

Values are mean ± SD, *n* (%), or median [interquartile range] unless otherwise stated. BMI = body mass index; CAD = coronary artery disease; MI = myocardial infarction; NSTEMI = non–ST-segment elevation myocardial infarction; PCI = percutaneous coronary intervention; QCA = quantitative coronary angiography; STEMI = ST-segment elevation myocardial infarction

**Table 2:** Coronary angiography evaluation of the derivation dataset and validation dataset.

	Derivation Set	Validation Set	*p*-Value
No. of patients	729	399	
Combined CAD			0.584
None	69 (9.47)	48 (12.03)	
1-vessel disease	134 (18.38)	82 (20.55)	
2-vessels disease	197 (27.02)	103 (25.81)	
3-vessels disease	260 (35.67)	134 (33.58)	
LM disease	3 (0.41)	0 (0.00)	
LM + 1-vessel disease	5 (0.69)	3 (0.75)	
LM + 2-vessels disease	11 (1.51)	3 (0.75)	
LM + 3-vessels disease	50 (6.86)	26 (6.52)	
SYNTAX score	14.50 [7.00, 21.50]	14.00 [7.00, 21.25]	0.674
LM ectasia	73 (10.01)	58 (14.54)	0.030
LAD ectasia	299 (41.02)	174 (43.61)	0.435
LCX ectasia	266 (36.49)	164 (41.10)	0.144
RCA ectasia	454 (62.28)	232 (58.15)	0.195
diffuse dilation	401 (55.01)	194 (48.62)	0.046
Markis classification			0.040
Type I	42 (5.76)	30 (7.52)	
Type II	161 (22.09)	83 (20.80)	
Type III	197 (27.02)	81 (20.30)	
Type IV	329 (45.13)	205 (51.38)	
MAXD	5.39 [4.59, 6.16]	5.54 [4.69, 6.30]	0.038
MAXD ratio	1.74 [1.59, 1.95]	1.74 [1.58, 2.01]	0.535
MAXL	31.07 [12.66, 62.39]	28.68 [12.15, 56.15]	0.190
MAXL ratio	0.36 [0.14, 0.69]	0.31 [0.13, 0.61]	0.090
contrast agent stasis	192 (26.34)	100 (25.06)	0.692
thrombus in dilated segment	14 (1.92)	6 (1.50)	0.786
calcification in dilated segment	47 (6.45)	28 (7.02)	0.808

Values are mean ± SD, *n* (%), or median [interquartile range] unless otherwise stated. CAD = coronary artery disease; LAD = left anterior descending artery; LCX = Left circumflex artery; LM = left main; MAXD = maximum diameter of dilated lesion; MAXD ratio = maximum diameter dilation ratio (ratio of max diameter to reference diameter); MAXL = maximum length of dilated lesion; MAXL ratio = maximum length ratio (ratio of lesion length to vessel length); RCA = right coronary artery; SYNTAX = Synergy Between PCI With Taxus and Cardiac Surgery.

**Table 3:** The prediction model based on multivariable Cox regression.

Variables	Hazard Ratio (95% CI)	*p*-Value
Age	1.037 (1.010, 1.065)	0.007
History of prior PCI	1.980 (1.091, 3.595)	0.025
NT-proBNP (Each increase of 500 ng/L)	1.528 (1.031, 2.264)	0.035
Increased hsCRP(>3 mg/L)	2.335 (1.325, 4.114)	0.003
QCA characteristic classification	-	0.011
Type 1	-	-
Type 2	1.745 (0.520, 5.854)	0.368
Type 3	1.829 (0.632, 5.294)	0.261
Type 4	3.703 (1.543, 8.888)	0.003

CI = confidence interval; HR = hazard ratio; hsCRP = high sensitivity C-reactive protein; NT-proBNP = N-terminal pro-brain natriuretic peptide; PCI = percutaneous coronary intervention; QCA = quantitative coronary angiography.

## Data Availability

The data presented in this study are available on request from the corresponding author. The data are not publicly available due to privacy and ethical restrictions.

## Supplementary Materials

The following are available online at https://www.mdpi.com/article/10.3390/jcdd8120186/s1. Table S1. Coronary artery reference diameter in 231 age-sex-matched angiographically normal subjects. Table S2: Collected clinical variables. Table S3. Correlation coefficient matrix of the candidate variables. Figure S1. Reported CAE in Fuwai hospital during 2009 to 2015. Figure S2. Schoenfeld residual test of the prediction model. Figure S3. Restricted cubic splines suggested hsCRP > 3 mg/L as an inflection point for hazard ratio.

## Appendix A

**Table A1:** Univariable Cox regression in derivation set.

Variables	*p* Value	Hazard Ratio (95% CI)
**Continuous Variable**		
Age	0.011	1.04 (1.01–1.06)
BMI	0.981	1.00 (0.97–1.03)
LVEF	0.107	0.98 (0.95–1.01)
LVID	0.143	1.03 (0.99–1.08)
D-dimer	0.616	1.09 (0.77–1.54)
Serum creatinine	0.512	1.00 (0.99–1.02)
eGFR	0.966	1.00 (0.99–1.01)
TC	0.847	1.02 (0.81–1.29)
TG	0.578	0.92 (0.68–1.24)
LDL-C	0.867	0.98 (0.73–1.30)
HDL-C	0.750	1.18 (0.43–3.27)
NT-proBNP (increase of 500 ng/mL)	0.001	1.77 (1.25–2.52)
hsCRP	0.007	1.08 (1.02–1.15)
ESR	0.608	1.00 (0.99–1.02)
SYNTAX score	0.399	1.01 (0.99–1.04)
MAXD	0.736	1.01 (0.95–1.08)
MAXD ratio	0.833	1.03 (0.81–1.30)
MAXL	0.165	1.01 (1.00–1.01)
MAXL ratio	0.061	2.14 (0.97–4.74)
**Categorical Variable**		
Male	0.263	1.69 (0.67–4.26)
MI at enrollment	0.815	1.09 (0.54–2.17)
Prior MI	0.188	1.47 (0.83–2.61)
Prior PCI	0.116	1.59 (0.89–2.85)
Diabetes	0.668	1.15 (0.61–2.15)
Hypertension	0.951	1.02 (0.57–1.82)
Dyslipidemia	0.964	0.99 (0.56–1.74)
Peripheral arterial disease	0.795	1.30 (0.18–9.41)
Family history of CAD	0.513	1.27 (0.62–2.61)
Current smoker	0.828	1.07 (0.60–1.89)
SYNTAX Score Level	-	-
low (≤22)	-	-
mid (>22 and ≤32)	0.211	0.58 (0.25–1.36)
high (>32)	0.788	1.17 (0.36–3.79)
Severe CAD (LM or 3-vessels disease)	0.332	1.31 (0.76–2.27)
LM ectasia	0.621	1.24 (0.53–2.91)
LAD ectasia	0.982	1.01 (0.58–1.76)
LCX ectasia	0.628	0.87 (0.48–1.55)
RCA ectasia	0.697	1.12 (0.63–1.99)
Diffuse dilation (MAXL ratio ≥ 1/3)	0.022	2.02 (1.11–3.69)
Markis Classification	-	-
Type I	-	-
Type II	0.179	4.00 (0.53–30.29)
Type III	0.160	4.22 (0.57–31.53)
Type IV	0.485	2.05 (0.27–15.48)
Contrast agent stasis	0.267	1.39 (0.78–2.49)
Thrombus in dilated segment	0.996	0.00 (0.00–Inf)
Calcification in dilated segment	0.500	0.61 (0.15–2.53)
LVEF < 50%	0.155	1.78 (0.80–3.96)
LVID > 55 mm	0.342	1.40 (0.70–2.79)
HsCRP > 3 mg/L	0.002	2.36 (1.36–4.09)
D-dimer > 0.5 mg/L	0.145	1.67 (0.84–3.34)
MAXD ≥ 5 mm	0.016	2.27 (1.16–4.42)
QCA Characteristic Classification	-	-
Type 1	-	-
Type 2	0.341	1.78 (0.54–5.83)
Type 3	0.278	1.80 (0.62–5.18)
Type 4	0.007	3.31 (1.38–7.92)

BMI = body mass index; CAD = coronary artery disease; CI = confidence interval; eGFR = estimated glomerular filtration rate; ESR = erythrocyte sedimentation rate; HDL-C = high density lipoprotein cholesterol; hsCRP = high sensitivity C-reactive protein; LAD = left anterior descending artery; LCX = Left circumflex artery; LDL-C = low density lipoprotein cholesterol; LM = left main artery; LVEF = left ventricular ejection fraction; LVID = left ventricular internal dimension; MAXD = maximum diameter of dilated lesion; MAXD ratio = maximum diameter dilation ratio (ratio of max diameter to reference diameter); MAXL = maximum length of dilated lesion; MAXL ratio = maximum length ratio (ratio of lesion length to vessel length); MI = myocardial infarction; PCI = percutaneous coronary intervention; NT-proBNP = N-terminal pro-brain natriuretic peptide; TC = serum total cholesterol; TG = serum total triglycerides; RCA = right coronary artery; SYNTAX = Synergy Between PCI With Taxus and Cardiac Surgery.
