# Supplementary material for: Development and Validation of a Nomogram for Predicting the Risk of Adverse Cardiovascular Events in Patients with Coronary Artery Ectasia
Source: J Cardiovasc Dev Dis. 2021 Dec 14;8(12):186. doi: 10.3390/jcdd8120186 (PMC8708195; doi:10.3390/jcdd8120186)

SUPPLEMENTARY MATERIAL

Table S1. Coronary artery reference diameter in 231 age-sex-matched angiographically normal subjects.

Table S2. Collected clinical variables.

Table S3. Correlation coefficient matrix of the candidate variables.

Figure S1. Reported CAE in Fuwai hospital during 2009 to 2015.

Figure S2. Schoenfeld residual test of the prediction model.

Figure S3. Restricted cubic splines suggested hsCRP>3mg/L as an inflection point for hazard ratio.

Table S1. Coronary artery reference diameter in 231 age-sex-matched angiographically normal subjects.

Coronary artery segment	reference diameter (mm)
Right coronary artery	
Proximal	3.7±0.6
Middle	3.1±0.5
Distal	2.8±0.5
Posterior descending	1.7±0.4
Posterolateral branch	1.9±0.4
Left anterior descending artery	
Proximal	3.2±0.6
Middle	2.8±0.5
Distal	2.0±0.4
Left circumflex artery	
Proximal	3.1±0.6
Distal	2.4±0.4
Obtuse marginal	1.9±0.5
Left main	4.4±0.6

Values are mean ± SD.

Table S2. Collected clinical variables.

Demographic data and medical history	Age, gender, BMI, Clinical presentation, prior MI, prior PCI, hypertension, diabetes, dyslipidemia, peripheral artery disease, family history of coronary heart disease, smoking
Laboratory test (at baseline and before coronary angiography)	white blood cell count, hemoglobin concentration, platelet count, serum creatinine, TC, TG, HDL-C, LDL-C, HbA1c, hsCRP, ESR, D-dimer, NT-proBNP.
Echocardiography	LVEF, LVID.

Table S3. Correlation coefficient matrix of the candidate variables

Variables	age	prior PCI	LVEF	LVID	hsCRP >3mg/L	NT-proBNP	MAXD≥5mm	MAXL ratio≥1/3	D-dimer＞0.5mg/L
age	1.000	-0.010	0.047	-0.179	-0.038	0.079	-0.121	-0.100	0.178
prior PCI	-0.010	1.000	-0.099	0.057	-0.078	-0.115	-0.028	-0.022	0.025
LVEF	0.047	-0.099	1.000	-0.426	-0.068	-0.241	-0.108	-0.079	-0.077
LVID	-0.179	0.057	-0.426	1.000	0.076	0.161	0.181	0.156	0.014
hsCRP >3mg/L	-0.038	-0.078	-0.068	0.076	1.000	0.167	0.032	0.019	0.092
NT-proBNP	0.079	-0.115	-0.241	0.161	0.167	1.000	0.034	0.046	0.084
MAXD≥5mm	-0.121	-0.028	-0.108	0.181	0.032	0.034	1.000	0.360	-0.023
MAXL ratio≥1/3	-0.100	-0.022	-0.079	0.156	0.019	0.046	0.360	1.000	0.042
D-dimer＞0.5mg/L	0.178	0.025	-0.077	0.014	0.092	0.084	-0.023	0.042	1.000

Figure S1. Reported CAE in Fuwai hospital during 2009 to 2015.

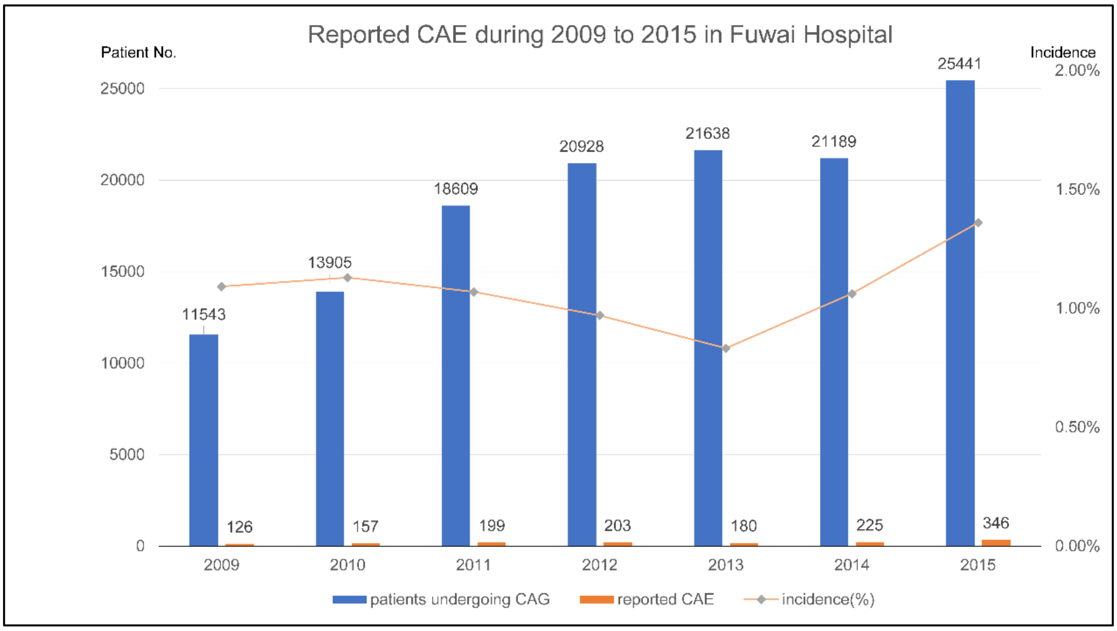

Figure S2. Schoenfeld residual test of the prediction model.

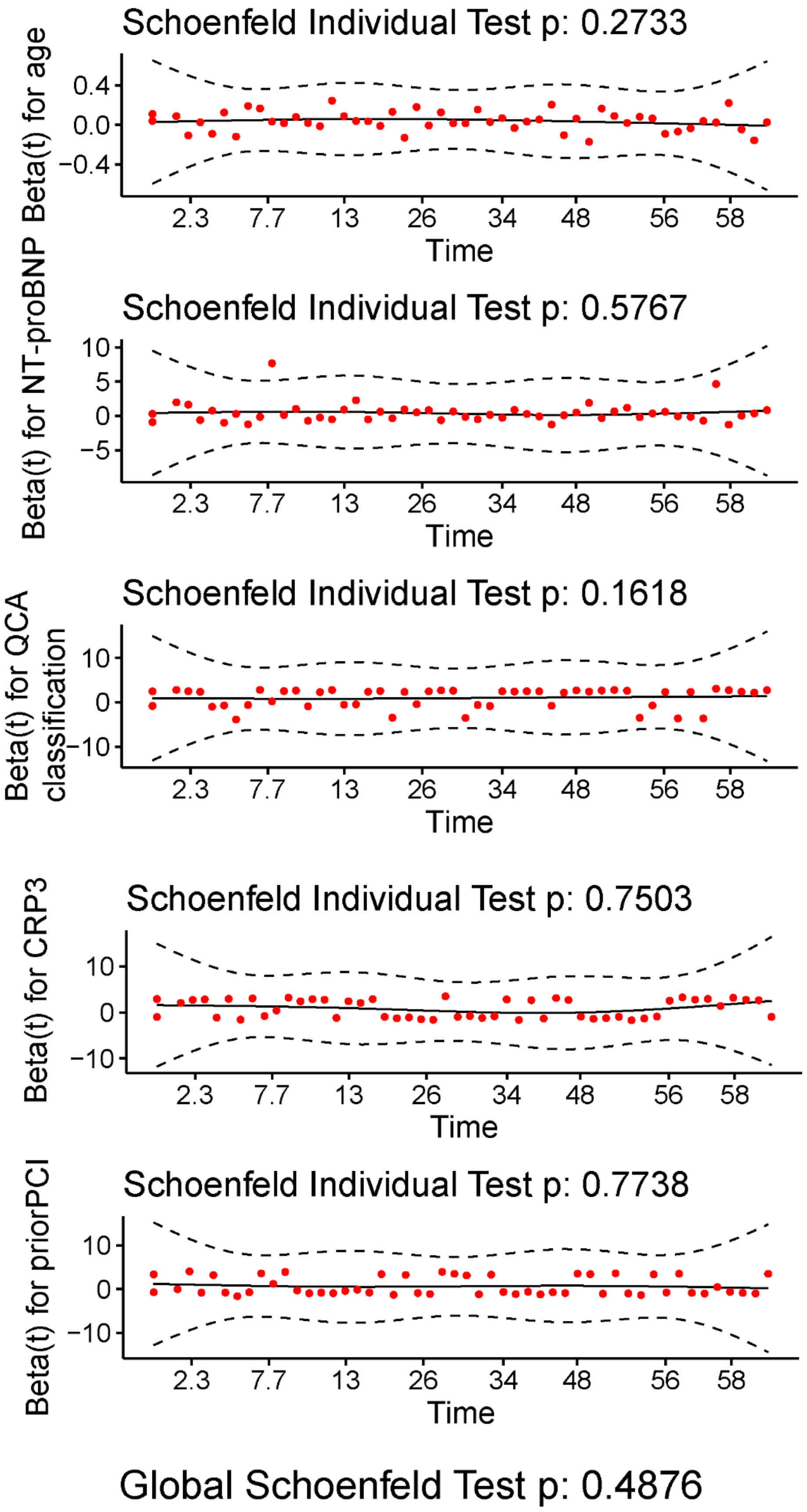

Figure S3. Restricted cubic splines suggested hsCRP>3mg/L as an inflection point for hazard ratio.

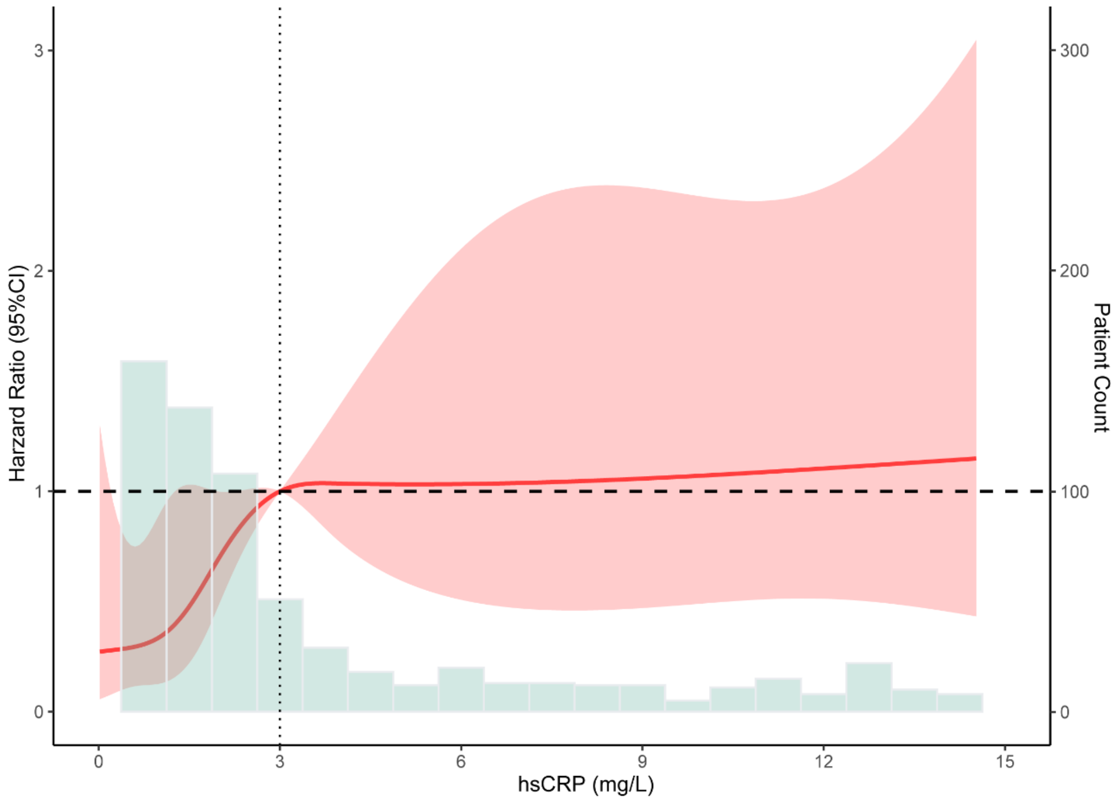

Supplement: Supplementary file 1 [file jcdd-08-00186-s001.zip › jcdd-1477758-supplementary.pdf]
